# Feature Extraction Approach for Speaker Verification to Support Healthcare System Using Blockchain Security for Data Privacy

**DOI:** 10.1155/2022/8717263

**Published:** 2022-07-25

**Authors:** Shrikant Upadhyay, Mohit Kumar, Ashwani Kumar, Ramesh Karnati, Gouse Baig Mahommad, Sara A. Althubiti, Fayadh Alenezi, Kemal Polat

**Affiliations:** ^1^Department of Electronics & Communication Engineering, Cambridge Institute of Technology, Ranchi 834001, India; ^2^Department of Computer Science & Engineering, Cambridge Institute of Technology, Ranchi 834001, India; ^3^Department of Computer Science & Engineering, Sreyas Institute of Engineering and Technology, Hyderabad 500068, India; ^4^Department of CSE, Vardhaman College of Engineering, Hyderabad, India; ^5^Department of Computer Science, College of Computer and Information Sciences, Majmaah University, Al-Majmaah 11952, Saudi Arabia; ^6^Department of Electrical Engineering, College of Engineering, Jouf University, Saudi Arabia; ^7^Department of Electrical and Electronics Engineering, Faculty of Engineering, BoluAbantIzzetBaysal University, Bolu, Turkey

## Abstract

Speech is one form of biometric that combines both physiological and behavioral features. It is beneficial for remote-access transactions over telecommunication networks. Presently, this task is the most challenging one for researchers. People's mental status in the form of emotions is quite complex, and its complexity depends upon internal behavior. Emotion and facial behavior are essential characteristics through which human internal thought can be predicted. Speech is one of the mechanisms through which human's various internal reflections can be expected and extracted by focusing on the vocal track, the flow of voice, voice frequency, etc. Human voice specimens of different ages can be emotions that can be predicted through a deep learning approach using feature removal behavior prediction that will help build a step intelligent healthcare system strong and provide data to various doctors of medical institutes and hospitals to understand the physiological behavior of humans. Healthcare is a clinical area with data concentrated where many details are accessed, generated, and circulated periodically. Healthcare systems with many existing approaches like tracing and tracking continuously disclose the system's constraints in controlling patient data privacy and security. In the healthcare system, majority of the work involves swapping or using decisively confidential and personal data. A key issue is the modeling of approaches that guarantee the value of health-related data while protecting privacy and observing high behavioral standards. This will encourage large-scale perception, especially as healthcare information collection is expected to continue far off this current ongoing pandemic. So, the research section is looking for a privacy-preserving, secure, and sustainable system by using a technology called Blockchain. Data related to healthcare and distribution among institutions is a very challenging task. Storage of facts in the centralized form is a targeted choice for cyber hackers and initiates an accordant sight of patients' facts which will cause a problem in sharing information over a network. So, this research paper's approach based on Blockchain for sharing sufferer data in a secured manner is presented. Finally, the proposed model for extracting optimum value in error rate and accuracy was analyzed using different feature removal approaches to determine which feature removal performs better with different voice specimen variations. The proposed method increases the rate of correct evidence collection and minimizes the loss and authentication issues and using feature extraction based on text validation increases the sustainability of the healthcare system.

## 1. Introduction

The sharing of healthcare information in different institutions is quite complex, which requires strong agreement with the promising model as there is a remarkable increase in the exchange of medical data in the network [1]. The main reason not to share data in network is privacy worry [2], creating panic while sending information may give an ambitious choice [3].

Blockchain technology arises as a key technology in the digital uprising of various verticals like healthcare, supply chains, and automobile industry. Many techniques are available for information sharing either using extraction or Blockchain approach but the integration of both found to be less from data privacy point of view. Creating a stable and more secure platform using updated dispersal blockchain increases the potential of healthcare system and that motivates highly. Blockchain utilization for healthcare management of data creates advantages for doctors, patients, and healthcare institutes for proper management of medical records, payment and claims management, data validation, and financial auditing. This also permits healthcare units to restrict the unsanctioned user to access sensitive data and restrict for any modification to patient's data for its sustainability. The primary goal of such technology is to deploy by integrating other existing technology to make an efficient health fact system with infrastructure to fulfil the healthcare system necessity.

**Figure 1:**
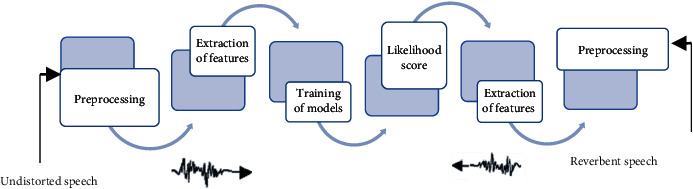
Method for speaker identification with undistorted and reverbent speech.

There is no comprehensive agreement on the particular infrastructure required to hold up such a job [4]. Knowledge of the meaning and structure of data is a primary requirement for the health while sending information among different institutions. Considering that facts are transferred securely and efficiently, these coordination businesses remain unreached to control facts' value [5]. For a far talking or speaking scenario, clean speech is somehow disturbed by reverberation. Due to this disturbance, an individual's speaker features also get distorted. There is a quarrel between the outline trial and instructed talk model getting recognized, which reduces the talked membrane's production [6]. To minimize this conflict, we present three steps involved methods for far speaker recognition from talk development, model, and attribute compensation opinion as depicted in Figure 1. Feature extraction from a speech has its own importance. Every person has its unique voice quality, voice throw, speaking style, talking, and pitch, and gathering the information from voice plays a crucial role for any model. GMM compared to other modeling approaches adds unique features because its training is relatively fast, and models can be updated and scaled to newly added speech easily. It is composed of a finite composition of multivariate Gaussian mixtures and calculates density function *δ*_*λ*_(*x*) as per:

(1) $$\begin{array}{c} \delta_{\lambda }\left(x\right)=\overset{P}{\underset{i=1}{\sum }}m_{i}\delta_{i}\left(x\right), \end{array}$$

where “*P*” is the Gaussian term and *m*_*i*_ is the prior probability of *i*^th^ D-variate density function given as:

(2) $$\begin{array}{c} \delta_{i}\left(x\right)=\frac{1}{{\left(2\pi \right)}^{Q/2}{\left|\Sigma_{i}\right|}^{1/2}\;}e^{-\left(1/2\right){\left(x-\mu_{i}\right)}^{T}}\overset{-1}{\underset{k}{\sum }}\left(x-\mu_{i}\right), \end{array}$$

where ∑_*i*_ and *μ*_*i*_ denotes the covariance matrix and mean vector of multidimensional Gaussian model [7]. Extraction of speech means taking some special characteristics which makes the identity unique for the person and distinguishing different from others in term of voice quality and known features will help to collect its original identity.

One of the major limitations of conventional GMM is its super vector presentation and successive factor model investigation which does not be counted of the fact that original acoustic characteristics [8]. The main contribution is the modeling of training and testing that verifies the said text using threshold level which collects the correct evidence. At the same time, the Blockchain contributes to monitor the sensitive data and maintain its security and privacy at each level while transferring in network.

## 2. Blockchain in Healthcare

A patient's medical status requires complete monitoring by the hospital and doctors for its better treatment while maintaining privacy and data security of sensitive data of patients to share them with medical institutes and leading hospitals for expert consultation for gathering better information about the related cases. As per accountability law and health insurance, law enforcement and many other public agencies access medical information legally. It is approximated that around 200 to 500 individuals may have the right to read the health records of any patient without any authentication and permission [9]. When information is distributed extensively and kept in multiple outbreaks, securing data is one of the more crucial issues. As per Ponemon Institute, in the year 2016, about 112 million information related to medical were negotiated and such break of data tempered and attacks raised by 162% in the year 2017 [10, 11]. Blockchain ideal in healthcare arises when there is a need for interoperability and security comes into the picture. Many healthcare gadgets and healthcare-related mobile applications boom with the evolution of and a large number of medical facts and important recorded data are transferred in today's life from various healthcare units. So, much facts traffic requires better management resources for its security and privacy. Blockchain is a technology that supports and provides a proper solution that will help to keep the recording. Secure and broadcast of medical information that maintains every patient's privacy to access their medical reports [12–22]. It enables an auditable path and creates transparency for individuals using their encryption key and unique credentials, and allows third parties to provide a grant to access individual medical records. It includes authority grants in terms of health competence, resource contributors, social care, and investigator to execute their medical facts and descriptions for the motive of healthcare delivery directly, statistical analysis, grant research, etc. Blockchain technology uses wise agreement to maintain records of patient interactions. The supplier generates a document, and once it is verified, the sufferer may have the right to view the reports [23–25]. An automatic notification will be generated after receiving new facts, and a coded indicator reflects to latest medical history. A chain is used to store the permission, which allows the patient to control access to their information. Areas where it is applicable will be vaccination records, blood trials, medicinal, therapeutic instruction, etc. [26–36].

**Example 1 .**

In the COVID-19 scenario for the healthcare system, it is challenging to acquire medical apparatus and medical supplies due to excessive demand. Issue of trust always arises for the collapse of stock sequence with authentic vendors. Various issues like the delivery deadline, payment, standards, customer verification, and cheating. This technology possibly assures such cases and maintains the credibility of distributors. So, by deploying such technology, support deadline and monitoring medicine requirements where there is excessive demand for medical needs. Blockchain technology adds transparency, security, and authentication by putting consent standards and protocols into a good flow of any aid. This is one of the primary concerns for the healthcare unit to become competent, and this can create a bridge between supply and demand chains for future advancement in the healthcare system.

**Example 2 .**

It can be suitable for wireless sensor network where nodes are dependent on each other for information sharing and dynamic nature make it unsecure for that data transmission.

**Example 3 .**

It can also be suitable for natural language processing for the text authentication in speech processing using different extraction approaches.

## 3. Facts Addition to Blockchain

**Figure 2:**
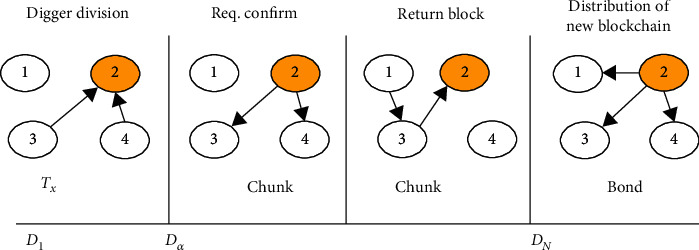
Blockchain phase involved in attaching chunk to healthcare system.

The fundamental occupation for addition of facts to the Blockchain is depicted in the figure below. Similar to bit coin concept, a bank is attached to Blockchain of uniform duration as this will be in bit coin identified by work function evidence. For network as depicted below, there is a uniform duration for generating a block. Inside this duration of block duration network goes through different phases of pursuit. The first phase is the agreement digger initialize at duration *D*_1_ and this undertaking transferred to the matching node, and this will continue until *D*_*α*_ when matching node no longer accepting updated undertaking for block. The phase of Blockchain involved in attaching chunk to care unit is depicted in Figure 2.

The coupling coefficient helps in attaching chunk to healthcare system that sign an agreement with differ and generates an effect of access to pass between the block. It can be modified by granting permission to each node and updating locally Blockchain for new dispersal. This grant permission to every node acts as a contributor once in undertaking to digitally mark to block, specifying that they inscribe actuality. The block new comes back to digger in return phase of signed block. The digger node added to the locally blockchain and in final phase dispenses the updated blockchain known as new dispersal blockchain. Algorithm first describes the way of generating new chunk and its addition to Blockchain.

## 4. Extraction and Interfacing

Extraction means to put chunk (block) to blockchain and similar concept like bitcoin but we try to follow some different technique where overall model bypass work model evidence where power calculation is spanned without putting some intrinsic value. Our aim is to create a network agreement by setting nodes to give evidence by reference of transaction with correct interpretation also at the same time node will verify the proof of potency. Such measures maintain consistency in Blockchain but also encourage better inferring among nodes [37]. Second algorithm discussed the steps involved by assigning a specific profile which is further compared with familiar set of admissible description.

If the description is identified, it will acknowledge with its confirmation using function Descp_Check and this will use the IP_URL to authenticate the server and the result request which will be response with IP_Outcome which will be further verified for conformity by Conf_Function.

Interfacing evidence does not need network to get arrangement with the set profiles which includes the indulge of the associated sets value. So, such agreement does not reach declaratively. Consensus in network is completely depending on process of human where network contestant can handshake and negotiate using both expression profession as well as specialist. Such collaboration is the necessary part for well familiar repository [38].

## 5. Digger Voting

For work evidence, digger runs to right to sum a chunk for Blockchain. Here, we put a sharing of digger same as multichain [39]. Adding these nodes will identify the duration of starting of chunk and who will be next digger. So, agreement may be sent directly without distribution to the whole network. Also, it assures that digger work requires to maintain the network smoothness dispense steadily.

Finally, by removing the contention of work evidence, we minimize and remove misused mathematical attempt. The third algorithm discussed the digger voting process. Recalling first algorithm where and step of collecting a chunk to Blockchain is for the node take part to enter. During this, allowing phases every node requires to deposit a random code in form of number for digger voting which is collected in line number 1 and hashed (#) with block to generate an updated new number.

The next digger becomes the node whose key (public) is nearest to that value and this process did two works

- Probability to become digger in network for any node will be 1/*N*_*n*_ (*N* = network, *n* = number of nodes) and
- Number that is generated randomly used for voting is planted by all contestant nodes in network and this will protect a node to issue a random code and selecting itself or with collaborator
- Randomly generated key provides a secure code to the participants

## 6. Improvement in Data Privacy

As discussed, facts security in terms of invisibility and privacy is the basic preference of any system. Security level in our proposed system based on several features includes special encryption, protection of privacy in term of keyword, and agreement in a smart way.

Encryption in Blockchain includes that whatever it stored is not a plain book public facts calculation for every node is predicted as encrypted using key shared in network and awareness facts should be encoded by activated node in network. Ease of fact exploration and availability secretly protected antonyms digging methods are used [40]. Following such techniques, outer institution may request for agreement from Blockchain to match some benchmark both for any query and agreement encryption. After this, the validation and identification process starts for the talker using Hindi speech for 50 speakers which was conducted using Cool tool and using MATLAB as a simulation tool.

## 7. Validation and Identification (Speaker)

**Figure 3:**
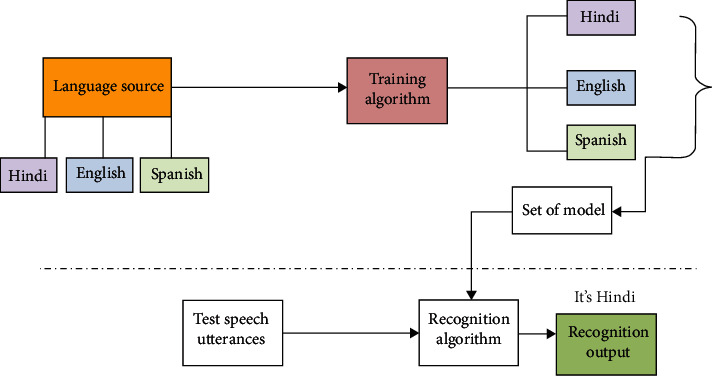
Phases involved in language recognition.

Taker recognition is very crucial to understand how a person understands the variables and voices that affect the hearing capabilities and the performance of listeners is firmly affected by speaking scenario and acoustic. Various investigations have been done to compare visual and aural talker recognition which reflects that aural inequity error is quite less than discrimination spectrogram error. In earlier, an attempt to a speaker recognition using automatic process was done that will help to analyze spoken utterance acoustically [41, 42]. Talker recognition spells the congruence of the talker where talker validation involves a method of rejecting or to obtain the identity of asserts users. Talker's validation is further divided into part which involves session training when model for user's talk is built up and real authentication is done. A model is first trained for new user's voice and be authenticated by mean of spectral investigation from which feature removal was done to create a talker model. Then, users' talk can be confirmed by weigh up against the instructed dataset of models. After comparison, the system will take decision where the claim's naming is one which is designed by teaching material available or not. The identification of different language is depicted in Figure 3.

### 7.1. Example: Trained and Untrained Talk

Both choral and trained signers have high cepstral compared to untrained singer and singing involves regular practice which involves scale, matching, notes, and its rhythms. The table shown below reflects that trained singer is found to be efficient compared to untrained in term of range, stability, control, etc. and have better control over loudness and pitch independent of frequency and intensity and nonsinger found with low proficiency in voice. In this research paper, we try to put all effort on information sharing with total control on facts by agreement concept between nodes and generation of random codes for authenticated users. Patient is your prime focus whose various health-related information shared to various medical institutions for proper analysis and fast recovery.

Privacy is one of the major concerns for any department and institutions. Healthcare-related data or facts shared through network are very challenging one. Sending of information using any network must have capability only to share information from source to destination and its security with privacy is real issue that has to be taken care of.

From Table 1, it is found that choral singer have a mean of 6.80 (±1.53) for comfort phonation and for reading it is 3.63 (±0.47). It shows that cepstral smoothened prominence cost of nonsinger are less than trained and group singers.

**Figure 4:**
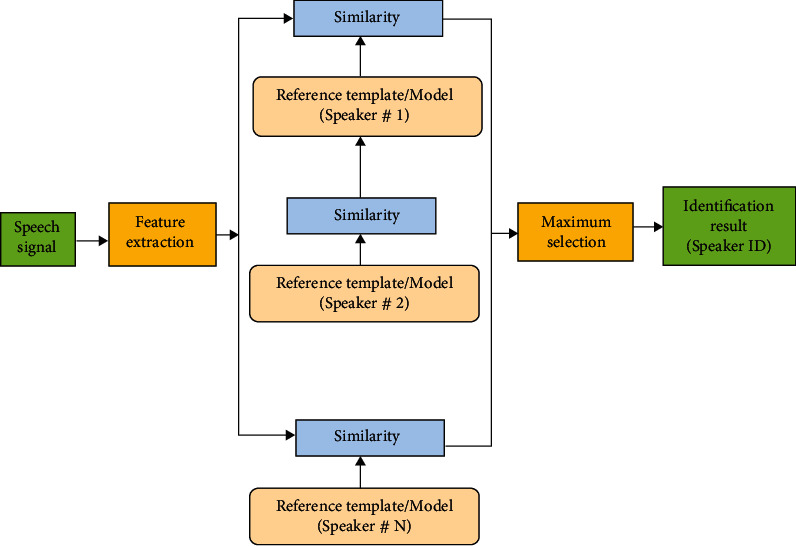
Recognition system consists of close set.

Recognition using speech in general used to include different way for discriminating human depending upon their voices. In recognition, a speech observation of unrecognized talker is analyzed and differentiates with all familiar talkers. The unrecognized talker is identified as talker whose model fit best the input declaration. The fundamental structure of talker recognition is depicted in Figure 4.

Probability concept involved for the generation of random codes for every user and mathematical complexity reduction algorithm for fast processing of incoming information. Keeping facts and records safely is a need of an hour and every healthcare institution focus on such technology for flawless exchange of information inside network system. Revolution and large amount of health-related gadgets involved for bulk amount of medical record transfer. So, security of shared data is very important and must have much more fighting capabilities against the cloud thief to make the facts clean.

**Figure 5:**
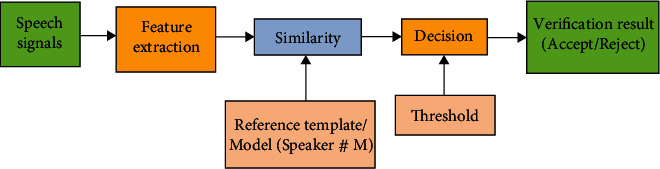
Basic structure for talker validation.

A closed set or unlocked set is termed for the talker recognition where in unlocked set assumption of test observation contain one the ‘N' register talker and in open set recognition there will be addition determination to identify whether verified observation was declared by one of ‘N' register talker or not i.e. *N*_+1_ determination. The basic structure of talker validation is depicted in Figure 5.

The aim of this structure is to allow or reject the talker who claim based on speech specimen. Matching among reference level and test is above certain threshold limit the assert is welcomed. A very high threshold arises a problem for fake to accept by system but always arises an issue of rejecting authenticate one. Low threshold make sure to accept the genuine one but also arise of accepting a fake one. Such issues can be solved by correlation metrics obtained from the speech spectra and feature vector modeling following parametric techniques. Here we also try to consider text-dependent stem as this will have high degree of control over speech conditions.

## 8. Feature Removal

The principle behind of this approach is to low down the average of total difference that arises between calculated speech and original speech over a definable period. The coefficient prediction can be used to find distinctive set which is generally 20 milliseconds lengthy. This approach is known as linear predictive technique [43].

Gain is an important parameter and time variable transfer function of filter is given as-

(3) $$\begin{array}{c} G\left(z\right)=\left(\frac{h}{1}-\sum P_{c}-s\right), \end{array}$$

where *P*_*c*_ = coefficients forecasts and *s* =1 to *n*.

The decision taking in term of analysis of every frame of unspoken and spoken signal was done using this approach. It also helps to employ pitch identification algorithm to detect best possible frequency of pitch and re-emphasis it as gain is varying frame to frame [43].

The next important approach is coefficient acoustic measure of mel frequency and considered to be a standard approach for feature removal. At least twenty coefficients are required in automatic recognition of speech where maximum ten to twelve coefficients are found to be sufficient for speech coding. This coding is quite sensitive to distortion or noise due to its spectral nature [44]. Procedure that employ facts in revolution of speech can be considered to minimize such issues as speech contains causal captivates.

Frequency nonlinear in nature used to approximate the mel component which is approximately below 1 kilohertz and above 1 kilohertz for frequency in logarithmic. Person auditory structure has minimum selective of frequency above 1 kilohertz. The feature of this approach follows energy of log filter storage to analyze the output [45–48]:

(4) $$\begin{array}{c} \left(t\right)=\ln\left(\overset{N-1}{\underset{n}{\sum }}\left|S_{t}{\left(n\right)}^{2}G_{t}\left(n\right)\right|\right)0\leq \text{t}<\text{T}, \end{array}$$

where *S*_*t*_(*n*) is the Fourier transform of *n*^th^ speech input frame and *G*_*t*_(*n*) is the response of *t*^th^ filter storage, *T* is total filter used, and *N* is size of window used to transfer. Then, at log energy, cosine transformation will be:

(5) $$\begin{array}{c} \left(t\right)=\overset{L-1}{\underset{l=0}{\sum }}R_{t}\left(n\right)\cos\left(\pi m\left(\frac{n-0.5}{T}\right)\right.\;0\leq t<T. \end{array}$$

So, to gather fluctuation in coefficient over duration 1^st^ and 2^nd^ coefficient differences are calculated as:

(6) $$\begin{array}{c} ∆\overset{⟶}{\left(f_{t}\right)}=\left(\overset{⟶}{f_{t}}\right)+2-\overset{⟶}{\;f_{t}}-2,\\ ∆∆\overset{⟶}{\left(f_{t}\right)}=∆\overset{⟶}{f_{t+1}}-∆\overset{⟶}{f_{t-1}}. \end{array}$$

Linear prediction approaches are used for the estimation of fundamental component of speech. In this method, input is first prehighlighted using filter of high pass and its transfer function will be [49]:

(7) $$\begin{array}{c} Z_{p}\left(n\right)=1-{bh}^{-1}. \end{array}$$

Now, windowing is carried out to minimize the discontinuities in each frame at edge using hamming to smooth side lobe and is given as:

(8) $$\begin{array}{c} v\left(w\right)=0.55-0.45\cos\left(2\pi \frac{m}{M}\right);0\leq m\leq M. \end{array}$$

The shape of spoken span basically determines the temper of produced sound and to model the vocal region we require transfer function in domain:

(9) $$\begin{array}{c} X\left(z\right)=\frac{H}{1-\sum_{n=1}^{r}b_{k}z^{-k}}, \end{array}$$

where *X*(*z*) represents the spoken span transfer function, *H* is the gain, and *b*_*k*_ is the throwback coefficients. Last phase which includes cepstral evaluation of sequenced speech was done.

(10) $$\begin{array}{c} p\left(n\right)=\frac{1}{2\pi }\int_{-\pi }^{+\pi }\ln v\left(w\right)e^{jnw}dw. \end{array}$$

Another method to estimate cepstral is linear perception cepstral coefficients. The feature of this approach follows energy of log filter storage to analyze the output:

(11) $$\begin{array}{c} R_{p}\left(t\right)=\ln\left(\overset{N-1}{\underset{n}{\sum }}\left|S_{t}{\left(n\right)}^{2}G_{t}\left(n\right)\right|\right)0\leq t<T. \end{array}$$

The linear scale equation will be:

(12) $$\begin{array}{c} LS=\sqrt{\frac{ns^{2}}{{\left(a_{1}\right)}^{2}+{\left(a_{2}\right)}^{2}+\cdots \cdots .+{\left(a_{n}\right)}^{2}}.} \end{array}$$

Suppose “*S*” is the target person's speech represented by *S* = {*s*_1_, *s*_2_, ⋯..*s*_*T*_} and “*V*” is the unobservable part of speech dependent represented by *V* = {*v*_1_, *v*_2_, ⋯..*v*_*T*_}; then for stastistically independent *S* and *V* probability, *P*(*x*_*t*_, *j*, *k*) can be calculated as [47]:

(13) $$\begin{array}{c} \text{P}\left(x_{t},j,k\right)=\int_{\infty }^{\infty }M\left(s;\underset{s,j}{\sum },\mu_{s,j}\right)M\left(x_{t}-s;\underset{v,k}{\sum },\mu_{v,k}\right), \end{array}$$

where ∑_*s*,*j*_ and ∑_*v*,*k*_ are weight covariance matrix and *μ*_*s*,*j*_ and *μ*_*v*,*k*_ are mean vector and *M* denotes the Gaussian density matrix.

A linear predictor of order “*p*” with prediction coefficient (*α*_*k*_) is defined as a system whose output is defined as [50–53]:

(14) $$\begin{array}{c} \hat{s}\left(n\right)=\overset{p}{\underset{k=1}{\sum }}\alpha_{k}S\left(n-k\right). \end{array}$$

The system function is *p*^th^ order polynomial and it follows:

(15) $$\begin{array}{c} P\left(z\right)=\alpha_{k}z^{–k}. \end{array}$$

The prediction error *e* (*n*) is defined as:

(16) $$\begin{array}{c} e\left(n\right)=s\left(n\right)-\hat{s}\left(n\right)=s\left(n\right)-\overset{p}{\underset{k=1}{\sum }}\alpha_{k}S\left(n-k\right). \end{array}$$

The transfer function of prediction error sequence is:

(17) $$\begin{array}{c} A\left(z\right)=1-\overset{p}{\underset{k=1}{\sum }}\alpha_{k}z^{–k}. \end{array}$$

## 9. Result and Discussions

**Figure 6:**
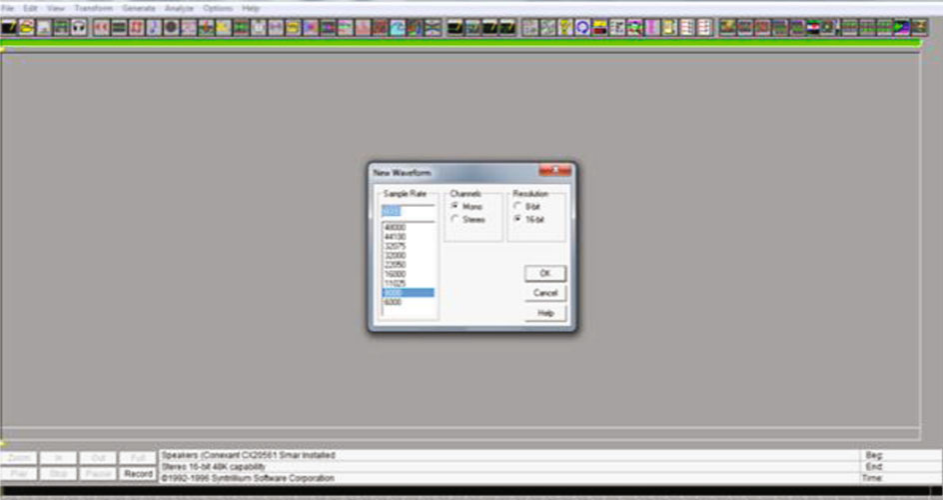
Parameters selection procedure as per specimen.

The dataset for fifty speakers with its value achieved is shown in Table 2. This dataset consists of fifty talked persons of different ages and group which help to match the pattern of the said voice and authentication will be done using this dataset. Text-dependent text was considered for the analysis of our research which will help the model to judge the quality of speech with better accuracy. The dataset for fifty speakers is shown in Tables 2 and 3 which represents the feature vectors for fifty speakers with their values from (a) to (c) and (d) denotes the parameters considered for its simulation with its channel, rate of sampling, repetition rate, etc. The parameter section procedure is shown in Figure 6.

This feature value obtained reflects the quality of talker that will help in validation of speakers using different dialects and measure the specimen best possible choice in term of its quality and accuracy. The error rate and efficiency rate are shown in Tables 4 and 5.

**Figure 7:**
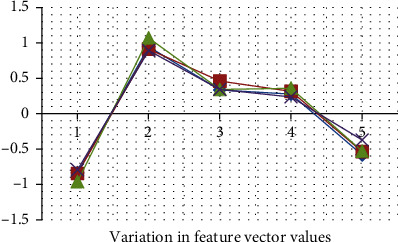
Feature vector variation for talkers.

**Figure 8:**
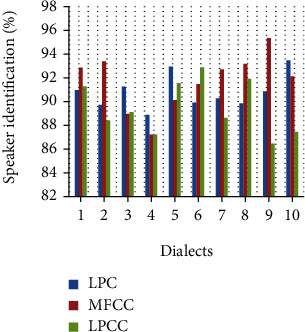
Efficiency of different feature extraction for speaker identification.

**Figure 9:**
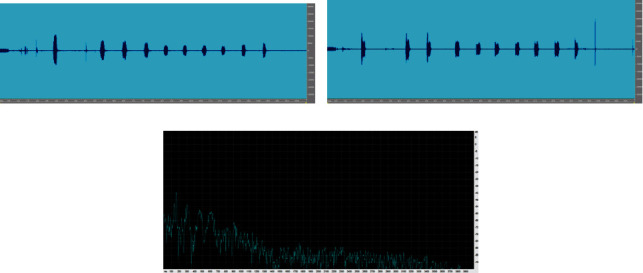
Variation in the spectrum for different feature of speech (a–c).

**Figure 10:**
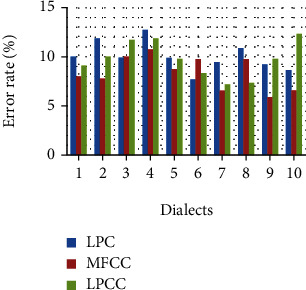
Error detection of different feature removal approach for Hindi dialects.

The feature vector variation for different talkers is depicted in Figure 7 where LPCC reflects the higher value compared to other said feature extraction. The efficiency of considered extraction is depicted in Figure 8 which is used for the identification of speakers. The error identification rate for the considered feature extraction is depicted in Figure 9 where LPC reflects the higher percentage of error rate compared to others. The variations of different features of talked speech are represented in Figures 9(a)–9(c). The error detection of different feature removal approach for Hindi dialects is shown in Figure 10.

Voice signal while sending network may degrade its original version so its original feature is very crucial to reach destination for matching. Calculating the efficiency rate of different feature removal approach of LPC, MFCC, and LPCC using Hindi dialects, we observed that the MFCCC will have better efficiency of 95.34% compared to LPC and LPCC with 92.95% and 92.895.

Training and validation under different simulation time up to 20 seconds have been conducted to calculate the error rate where we find that MFCC will have the least error rate of 5.90% compared to LPC and LPCC, i.e., 7.71% and 7.19%.

## 10. Conclusion

Data security and privacy are the primary concerns of this research article. Three algorithms play a crucial role by updating the node chain and adding it to the local Blockchain. The evidence collection procedure is also highlighted with some sort of agreement procedure to minimize the evidence loss and authentication construction, also putting systematic limitations on information access. A familiar set of admissible concepts is also added to differentiate the unknown group.

The confirmation function is used to authenticate the server. Healthcare vital record facts can be significantly protected after this validation is done, and finally voting approach is applied in the last algorithm to minimize the complex mathematical attempt and to make this algorithm fast which will also help to generate a new block of every time for the patients in term of probability. Whenever a sufferer wants to access their information, a random number is generated using this approach and protect to issue an arbitrary unauthorized code for users.

Such innovative agreement will provide more security to medical records and maintains a secure path in the chain supply process. Also, this verification process was done using three feature removal approach considering Hindi dialects which are text-dependent and validation was done in term of efficiency and error rate using a suitable model. Feature extraction is vital as information is transmitted using text or voice. If the agent is a choice, then MFCC will be the first choice for its better efficiency and minimum error rate. The integration of speech identification with the platform controls the talked dialects and gathers accuracy of high predictive, which is crucial for any system. This platform enhances the bright healthcare facility and provides a better way to exchange information to medical and research-related institutions for better care and evaluation of medical records. Blockchain maintains the security in data/facts while transmission creates a security layer for authentication of the correct user by extracting their voice quality using the feature extraction approach.

## Figures and Tables

**Algorithm 1:**
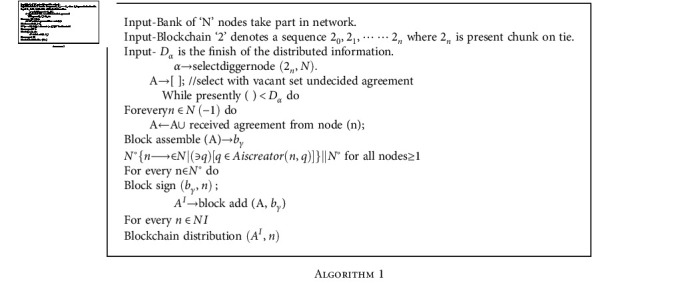


**Algorithm 2:**
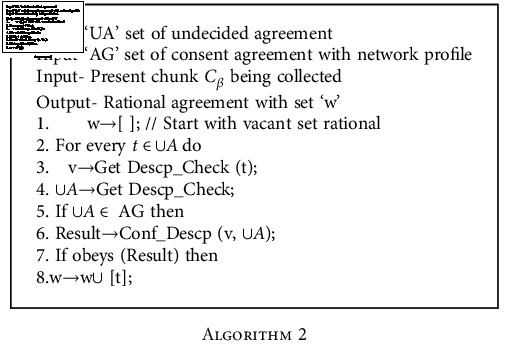


**Algorithm 3:**
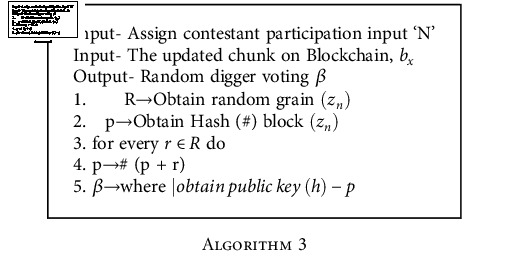


**Table 1:** Mean and deviation of different group.

Smoothened cepstral peak prominence	Group	Standard deviation	Mean
Sustained phonation	Choral singers	1.52	6.81
	Nonsingers	2.30	3.47
	Trained singers	1.66	7.10
	Overall	2.48	5.70

Reading task	Choral singers	0.48	3.62
	Nonsingers	0.45	2.20
	Trained singers	2.27	3.81
	Overall	1.44	3.17

**Table 2:** Dataset of 50 speakers.

D:\DB_50\0\00	D:\DB_50\0\01	D:\DB_50\0\02	D:\DB_50\0\03	D:\DB_50\0\04
D:\DB_50\0\05	D:\DB_50\0\06	D:\DB_50\0\07	D:\DB_50\0\08	D:\DB_50\0\09
D:\DB_50\1\00	D:\DB_50\1\01	D:\DB_50\1\02	D:\DB_50\1\03	D:\DB_50\1\04
D:\DB_50\1\05	D:\DB_50\1\06	D:\DB_50\1\07	D:\DB_50\1\08	D:\DB_50\1\09
D:\DB_50\2\00	D:\DB_50\2\01	D:\DB_50\2\02	D:\DB_50\2\03	D:\DB_50\2\04
D:\DB_50\2\05	D:\DB_50\2\06	D:\DB_50\2\07	D:\DB_50\2\08	D:\DB_50\2\09
D:\DB_50\3\00	D:\DB_50\3\01	D:\DB_50\3\02	D:\DB_50\3\03	D:\DB_50\3\04
D:\DB_50\3\05	D:\DB_50\3\06	D:\DB_50\3\07	D:\DB_50\3\08	D:\DB_50\3\09
D:\DB_50\4\00	D:\DB_50\4\01	D:\DB_50\4\02	D:\DB_50\4\03	D:\DB_50\4\04
D:\DB_50\4\05	D:\DB_50\4\06	D:\DB_50\4\07	D:\DB_50\4\08	D:\DB_50\4\09

**(a):** 

-0.836654	1.119128	-0.949380	0.946176	-0.008731
-0.846651	1.181489	-0.929819	0.911423	0.110117
-0.961810	1.390200	-1.052452	1.069067	-0.045332
-0.795792	1.283035	-1.010187	0.892773	-0.047438

**(b):** 

-0.607713	0.164727	-0.584664	-0.161334
-0.732032	0.258739	-0.537848	-0.110984
-0.748946	0.325589	-0.532755	0.015159
-0.557250	0.247957	-0.373091	-0.029203

**(c):** 

0.256184	0.334360	0.162019	-0.110454	0.276337
0.074978	0.460313	0.044143	-0.198783	0.314915
0.081630	0.343054	0.008088	-0.249718	0.358225
0.037462	0.342918	-0.066260	-0.160299	0.235376

**(d):** 

Parameters	Values
Talkers	0-9
Channel (single)	5KHz
Rate of sampling	8 bit (used for low noise)
Dialects	Text (Hindi)
Each speaker repetition rate	5 digits/minute

**Table 4:** Error rate in less acoustic scenario (noise).

Dialects	Train	Test	Feature extractions		
Hindi	(sec)	(sec)	LPC (%ER)	MFCC (%ER)	LPCC (%ER)
lnk	10	10	10.01	8.01	9.11
/kU; okn	10	5	11.88	7.77	10.02
gkWa	10	20	9.90	10.03	11.73
okyk	5	10	12.74	10.75	11.88
ebZ	5	5	9.88	8.75	9.78
vdcj	5	20	7.71	9.77	8.33
iz t	20	10	9.46	6.56	7.19
ia[kk	20	5	10.89	9.77	7.36
eka; k	20	20	9.22	5.90	9.79
vua	20	10	8.66	6.57	12.34

**Table 5:** Efficiency rate in (%) of individual feature extraction.

Hindi dialects	LPC (%)	MFCC (%)	LPCC (%)
Lnk	90.96	92.86	91.29
/kU; okn	89.73	93.38	88.43
GkWa	91.26	88.96	89.12
Okyk	88.89	87.23	87.23
EbZ	92.95	90.12	91.56
Vdcj	89.93	91.48	92.89
iz t	90.28	92.71	88.62
ia[kk	89.86	93.18	91.93
eka; k	90.86	95.34	86.46
Vua	93.48	92.14	87.46

## Data Availability

We can send the datasets at the request of the authors.
